# Are COVID-19 systematic reviews up to date and can we tell? A cross-sectional study

**DOI:** 10.1186/s13643-023-02253-x

**Published:** 2023-05-18

**Authors:** Steve McDonald, Simon L. Turner, Phi-Yen Nguyen, Matthew J. Page, Tari Turner

**Affiliations:** 1 Cochrane Australia, School of Public Health and Preventive Medicine, Monash University, Melbourne, 553 St. Kilda Road, Melbourne, VIC 3004 Australia; 2 Methods in Evidence Synthesis Unit, School of Public Health and Preventive Medicine, Monash University, Melbourne, 553 St. Kilda Road, Melbourne, VIC 3004 Australia

**Keywords:** COVID-19, Systematic reviews, Search reporting

## Abstract

**Background:**

COVID-19 led to a rapid acceleration in the number of systematic reviews. Readers need to know how up to date evidence is when selecting reviews to inform decisions. This cross-sectional study aimed to evaluate how easily the currency of COVID-19 systematic reviews published early in the pandemic could be determined and how up to date these reviews were at the time of publication.

**Methods:**

We searched for systematic reviews and meta-analyses relevant to COVID-19 added to PubMed in July 2020 and January 2021, including any that were first published as preprints. We extracted data on the date of search, number of included studies, and date first published online. For the search date, we noted the format of the date and where in the review this was reported. A sample of non-COVID-19 systematic reviews from November 2020 served as a comparator.

**Results:**

We identified 246 systematic reviews on COVID-19. In the abstract of these reviews, just over half (57%) reported the search date (day/month/year or month/year) while 43% failed to report any date. When the full text was considered, the search date was missing from 6% of reviews. The median time from last search to publication online was 91 days (IQR 63–130). Time from search to publication was similar for the subset of 15 rapid or living reviews (92 days) but shorter for the 29 reviews published as preprints (37 days). The median number of studies or publications included per review was 23 (IQR 12–40). In the sample of 290 non-COVID SRs, around two-thirds (65%) reported the search date while a third (34%) did not include any date in the abstract. The median time from search to publication online was 253 days (IQR 153–381) and each review included a median of 12 studies (IQR 8–21).

**Conclusions:**

Despite the context of the pandemic and the need to easily ascertain the currency of systematic reviews, reporting of the search date information for COVID-19 reviews was inadequate. Adherence to reporting guidelines would improve the transparency and usefulness of systematic reviews to users.

**Supplementary Information:**

The online version contains supplementary material available at 10.1186/s13643-023-02253-x.

## Background

Systematic reviews (SR) summarise evidence from primary studies with a view to informing decisions or guiding policy as they relate to a defined question or topic. How useful systematic reviews are in this regard may depend on several factors, including their methodological rigour and the extent to which they address questions that are important to end users. The timeliness of systematic reviews—how rapidly they are conducted and published—is another marker of usefulness. Systematic reviews that take many months, or even years, to conduct or spend a lengthy time in editorial review may be critically out of date by the time of publication [1].

During the COVID-19 pandemic timeliness became even more pressing as clinicians, guideline developers and others required up-to-date summaries of the latest evidence. As research from primary studies on COVID-19 emerged, an avalanche of systematic reviews followed. By May 2022, at least 4000 had been added to PubMed and over 7000 had been registered in PROSPERO. Several studies have identified deficiencies in the reliability of systematic reviews relevant to COVID-19 and highlighted less than optimal reporting [2–6].

One way that readers can navigate through the mass of systematic reviews on the same or similar topic, especially when evidence is accumulating rapidly, is to select the most recent review. This relies on the good reporting and timely conduct of reviews. The ability to assess currency depends on authors reporting the search date, ideally in the abstract of the review, which readers can quickly scan. Reporting guidelines, such as PRISMA [7] and PRISMA for Abstracts [8], and organisations which provide guidance in the conduct of systematic reviews, such as Cochrane [9] and JBI [10], have for many years recommended the inclusion of the search date (month and year) in the abstracts of systematic reviews. However, one study of 300 systematic reviews published between 2009 and 2011 showed that although 90% provided the date of search (month and year) in the main text of the paper, fewer than half (48%) stated this in the abstract [11].

In times of rapid evidence accrual, currency of reviews depends on how quickly they are conducted and published. Reviews are notoriously time-consuming—an analysis of 195 reviews from 2017 estimated the mean time to complete and publish a review was 67 weeks [12]. In contrast, early in the pandemic, review conduct and publication was rapid, with many journals fast-tracking COVID-19 publications [13]. An analysis of 88 COVID-19 systematic reviews published before June 2020 found that over 70% were conducted within 3 weeks, and half were published within 3 weeks of submission [3]. Given the accelerated pace of research publication output relating to COVID-19, being able to easily identify how up to date reviews are is important for users of systematic reviews.

### Objectives

Our primary objective was to evaluate how easily the currency of COVID-19 systematic reviews published in the first 12 months of the pandemic could be determined, as measured by where in the paper review authors reported the search date and how completely this information was reported (i.e., day/month/year or month/year only). Secondary objectives were to evaluate how up to date these systematic reviews were, as measured by the number of days from date of search to publication online, and to ascertain the number of studies or publications included per review. As a comparison, we included a contemporary sample of non-COVID-19 systematic reviews.

## Methods

### Data search and inclusion criteria

We searched PubMed for all systematic reviews and meta-analyses relevant to COVID-19 added to PubMed in July 2020 and January 2021 (see Additional file 1). The selection of these 2 months was linked to our involvement in the Australian National COVID-19 Clinical Evidence Taskforce guidelines [14]. During the first year of the pandemic when we were screening systematic reviews relevant to the guideline, we noticed how many failed to provide information about the search date. For this study, we adopted inclusive eligibility criteria, such that any self-described systematic review or meta-analysis of studies addressing any type of question (intervention effects, diagnostic test accuracy, prognostic factors, etc.) relevant to informing clinical practice or policy in relation to COVID-19 was included. SRs published in languages other than English were eligible, as were scoping, rapid or living reviews. Exclusion criteria were review protocols and reviews of animal studies, reviews without abstracts and reviews where it was not possible to determine the online publication date.

### Review selection and data extraction

Two authors (SM and TT) independently screened records in Covidence, with discrepancies resolved through discussion. One author (SM) extracted the following data from the PubMed record and the full-text report of each eligible review: date first published online, date added to PubMed, date of search, and number of included studies. In addition, we noted the format of the search date (i.e., day/month/year, month/year only, or no date) and where in the paper this was reported. For reviews that did not report the complete search date (i.e., day/month/year) in the abstract, we checked if this information was reported in the full text of the review, including in any supplementary files or Appendices. Data from a 10% random sample of reviews were independently checked by a team member (not involved in the study) to identify any discrepancies. (No discrepancies were found, and no further data checks were made.) Reviews were also assigned broad categories, such as diagnosis or treatment.

To determine whether any of these reviews had first been published as preprints, each review was checked against the NIH COVID-19 Portfolio database [15] using key bibliographic information, such as the first author and title words. If reviews had preprint versions, we noted the dates posted to the respective preprint server.

A random sample of 290 non-COVID-19 systematic reviews of intervention effects added to PubMed in November 2020 served as a comparison. (Ten reviews were excluded from the original sample of 300 reviews, either because they were COVID-19 reviews, or the online publication date could not be determined.) This sample was derived for a separate project [16] and used stricter inclusion criteria—systematic reviews had to have clearly stated objective(s), report the sources searched, include an assessment of risk of bias, and contain at least one pairwise meta-analysis. For each review, one author (SM) extracted the date of search (including the format and where reported) and date the review was published online. A second author (P-YN) extracted the number of included studies.

### Data analysis

We used descriptive statistics to summarise the completeness of reporting of the search date across the sample of COVID-19 and non-COVID-19 systematic reviews. Excel was used to calculate the number of days between the search date and the date the review was published and added to PubMed. These data were also calculated for the subset of reviews first published as preprints. We also stratified the analysis by the two 1-month periods for the COVID-19 sample (i.e., Jul 2020 and Jan 2021). Differences in medians (with associated confidence intervals) between the COVID-19 and non-COVID-19 samples were assessed using quantile regression analysis in Stata SE, version 16.1 (StataCorp).

## Results

The search of PubMed for July 2020 and January 2021 retrieved 340 records, of which 74 were excluded as not relevant to informing practice or policy for COVID-19. During the data extraction phase, we excluded an additional 20 reviews, either because it was not possible to determine the date the reviews were first published online (*n* = 11) or because the reviews had no abstract in PubMed (*n* = 9). In total, 246 COVID-19 systematic reviews were included in the analysis.

### Characteristics of COVID-19 SRs

The vast majority of reviews (93%) had *systematic* or *meta-analysis* in the title, and 17 (7%) had *living* or *rapid.* From our search of the NIH COVID-19 Portfolio, we determined that 34 reviews (14%) had been published as preprints (19 medRxiv; 8 Research Square; 4 SSRN; 3 preprints.org). The reviews were broadly categorised as treatment (13%), diagnosis (8%), prognosis (19%), epidemiology (50%), and prevention (10%).

The 246 SRs were published in 176 journals: 142 journals published one SR; 22 published two SRs; four published three SRs (BMJ Open; Crit Care; Hepatol Int; J Matern Fetal Neonatal Med); one published four SRs (J Clin Med); three published five SRs (Front Med; Int J Environ Res Public Health; PLoS One); two published six SRs (Cochrane Database Syst Rev; Diabetes Metab Syndr); and two published eight SRs (Int J Infect Dis; J Med Virol).

### Reporting the search date

#### COVID-19 SRs

**Fig. 1:**
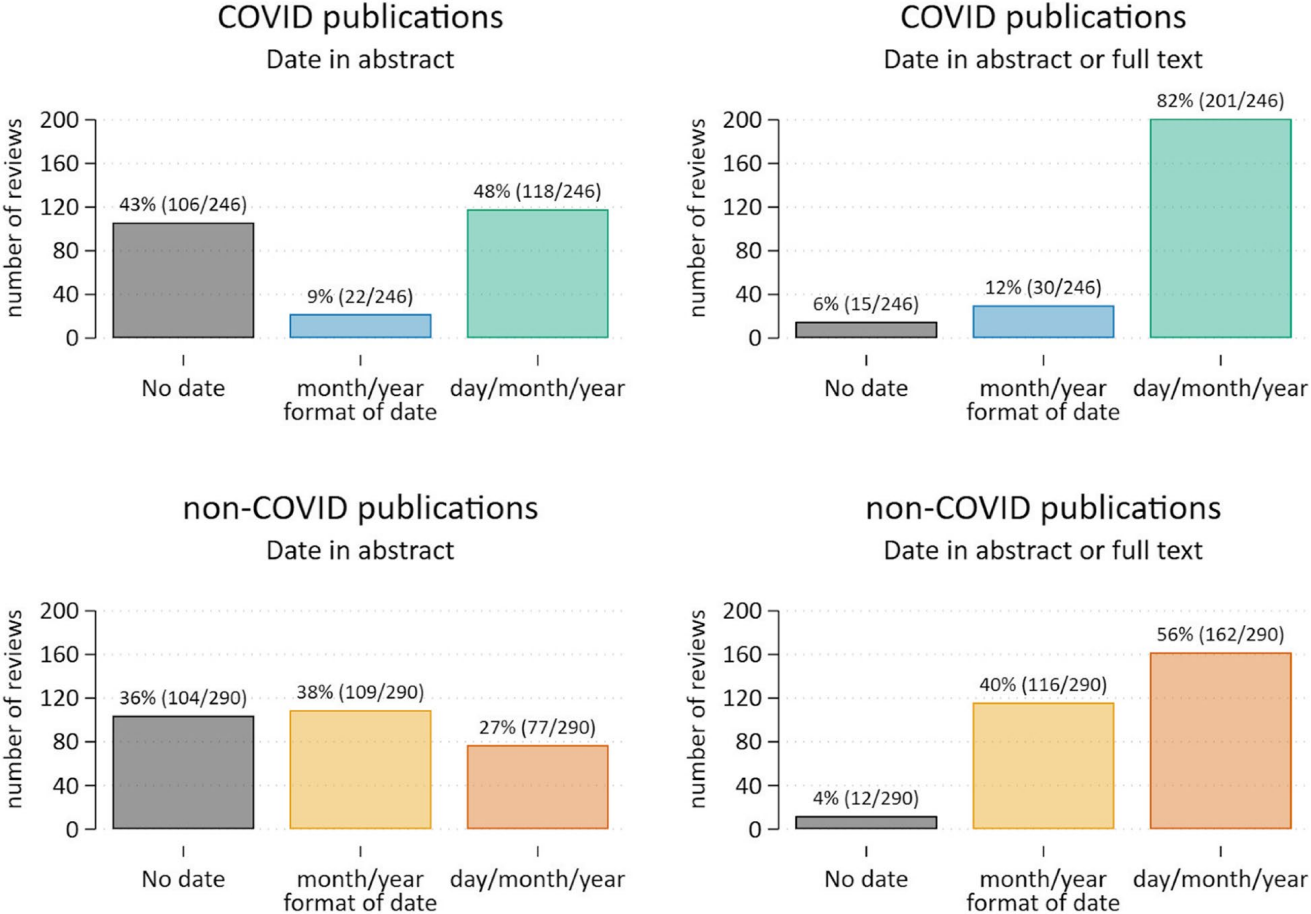
Reporting of the search date for COVID-19 and non-COVID-19 SRs

Of the 246 COVID-19 SRs, about half (48%; 118/246) reported the complete search date (i.e., day/month/year) in the abstract, 9% (22/246) reported the month/year only, and 43% (106/246) did not report any date in the abstract. (No meaningful difference was detected in reporting the search date in the abstract when comparing the July 2020 and January 2021 sample of reviews). Looking at the full review (including the abstract and any supplementary files), 82% (201/246) reported the complete search date, 12% (30/246) reported the month/year only, and 6% (15/246) reported no date (Fig. 1).

The PRISMA and PRISMA for Abstracts reporting guidelines [8, 17] require the inclusion of search date information in the abstract, yet even for the subset of 23 COVID-19 reviews which explicitly mentioned in the abstract of the review that the review was conducted following the PRISMA guidelines, over half (57%; 13/23) failed to report any search date information in the abstract.

#### Non-COVID SRs

For the sample of 290 non-COVID SRs, around a quarter (27%; 77/290) reported the complete search date in the abstract, 38% (109/290) reported the month/year only, 2% (6/290) reported the year only, and a third (34%; 98/290) did not report any date in the abstract. Looking at the full review (including the abstract and any supplementary files), 56% (162/290) reported the complete search date, 40% (116/290) reported the month/year only, one reported the year only, and 4% (12/290) reported no date (Fig. 1).

### Time from search to publication

#### COVID-19 SRs

**Fig. 2:**
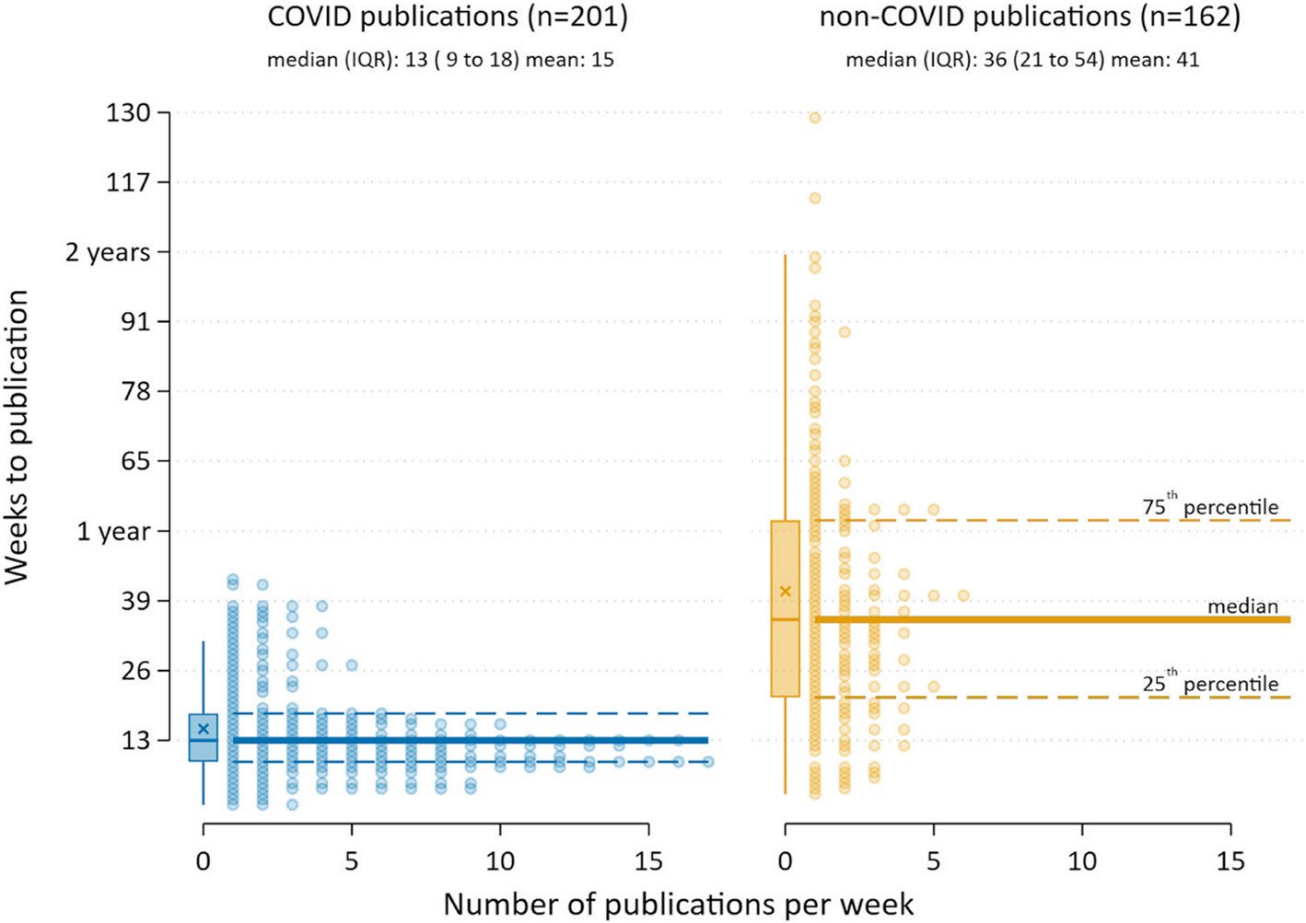
Time to publication from date of search for COVID-19 and non-COVID-19 SRs

Based on the 201 SRs that reported the complete search date, the median number of days from search to publication online was 91 (IQR 63–130; range 11–305), equivalent to 13 weeks (Fig. 2). Around a quarter (27%) of SRs were published within 2 months of completing the search, while 17% took longer than 6 months to be published. For the living or rapid reviews that reported the complete search date (15 out of 17), the median number of days from search to publication was almost identical (92 days (IQR 54–117)). Comparing the two periods, the median time to publication increased by 8 weeks from 72 days (IQR 54–92) in July 2020 to 130 days (IQR 90–208) in January 2021.

For the SRs first published as preprints, 85% (29/34) reported the complete date in either the abstract or full text. The median time from date of search to publication on the preprint server was considerably shorter (37 days (IQR 19–81; range 9–197)).

The median number of days from search to inclusion in PubMed was 98 (IQR 70–165; range 15–307), equivalent to 14 weeks. Three-quarters of SRs appeared in PubMed within 2 weeks of publication online.

#### Non-COVID-19 SRs

Based on the 162 SRs that reported the complete search date, the median number of days from search to publication online was 253 (IQR 153–381; range 21–1831), equivalent to 36 weeks. One in 10 SRs (9%) were published within 2 months of completing the search, while a third (32%) took 6 months to be published. Forty-seven (29%) SRs were published at least 12 months after the search was conducted (Fig. 2).

There is a clear difference in median time to publication from search date between the COVID and non-COVID cohorts (159 days fewer in the COVID cohort, 95% confidence interval (CI) 133 to 185).

### Included studies/publications

**Fig. 3:**
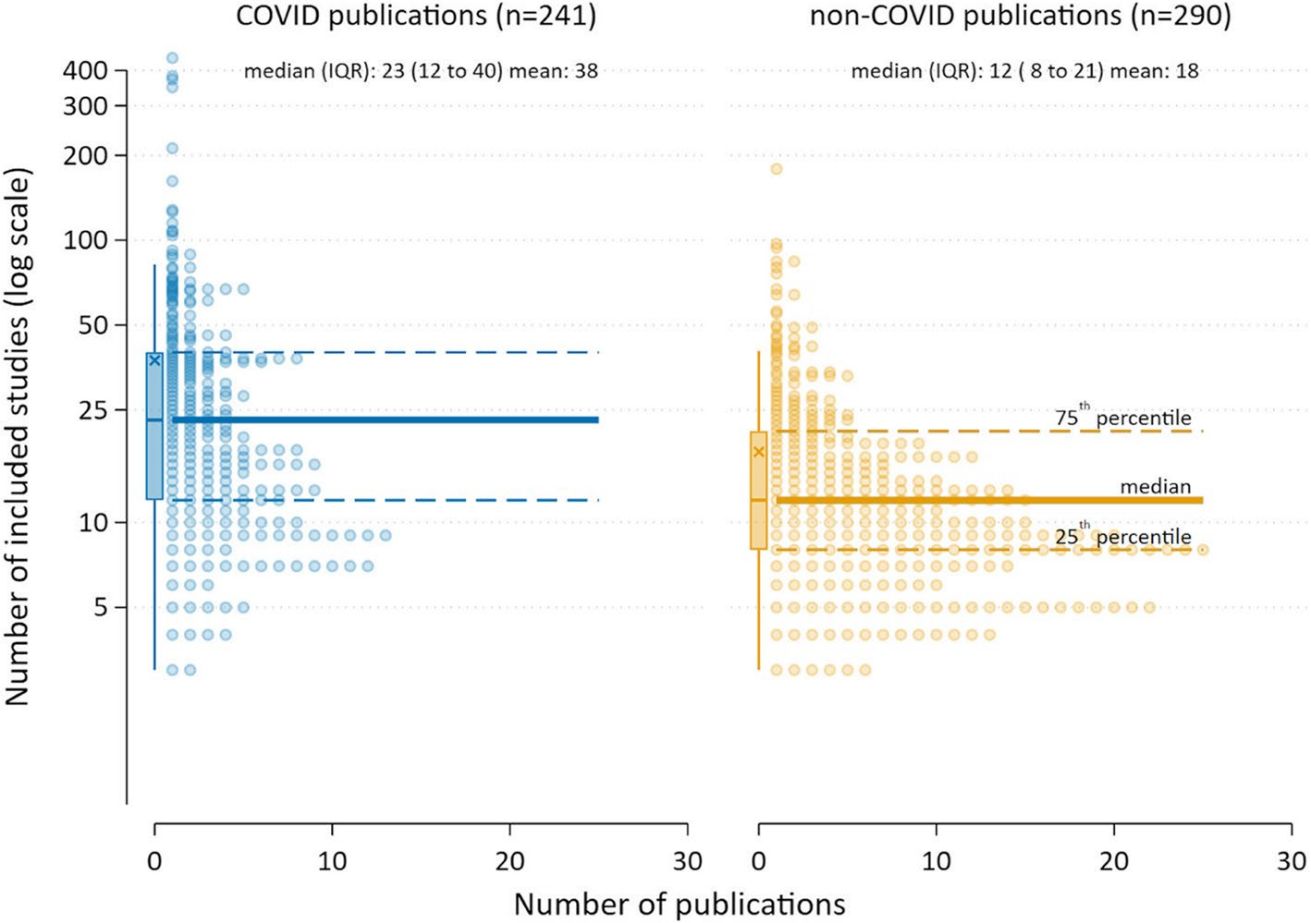
Number of included studies for COVID-19 and non-COVID-19 SRs

We were able to extract the number of included studies/publications for 98% (241/246) of the COVID-19 SRs. The median number of studies or publications included per review was 23 (IQR 12–40; range 3–443). The median decreased from 25 for the July 2020 sample of SRs to 21 for the January 2021 sample. The 290 non-COVID-19 SRs included a median of 12 studies (IQR 8–21; range 3–179), 11 fewer (95% CI 8 to 14 fewer) than in the COVID cohort (Fig. 3).

## Discussion

Despite the context of the pandemic and the need for readers to easily ascertain the currency of systematic reviews, 43% of our sample of 246 COVID-19 SRs failed to report any information on the search date in the abstract, and 6% failed to report the search date anywhere in the review. This compared with 36% and 4% respectively for the contemporary sample of non-COVID-19 systematic reviews. Since the search date for systematic reviews is the critical indicator of review currency, the failure of a large proportion of COVID-19 reviews to report even the month and year of the search in the abstract is surprising, especially given how fast the evidence around COVID-19 was accruing.

Although several studies have highlighted deficiencies with reporting of COVID-19 systematic reviews when applying the AMSTAR-2 checklist [2–4], we are not aware of studies that have explicitly looked at reporting of the search date. Poor reporting may be attributed to several factors at play during the pandemic, including the speed at which reviews were conducted and prepared for submission, as well as expedited editorial processes in which usual checks may have been overlooked. Yet even reviews that claimed to adhere to PRISMA frequently failed to report any search date information in the abstract.

Our finding that 94% of COVID-19 reviews reported at least the partial search date (month/year) somewhere in the review is similar to the 96% reporting this information in the non-COVID-19 sample of reviews from November 2020, and compares favourably to two previous studies (from 2009 to 2011 and 2014) that found this information was reported in 90% and 85% of reviews [8, 18].

The requirements of the pandemic compelled systematic reviewers to conduct reviews at lightning speed and journals to accelerate peer review processes [13, 19]. Unsurprisingly, the median time from last search date to publication online was considerably shorter for the COVID-19 SRs compared with the non-COVID-19 SRs (3 versus 8 months). A study of SRs conducted in the first few months of the pandemic reported even faster median times (within 3 weeks for conduct and within 3 weeks from submission to publication) [3]. In another sample of nearly 300 SRs on COVID-19 published before November 2020, the median time from submission to acceptance was 33 days [2].

The 8-month time lag from search to publication for non-COVID-19 SRs was similar to a cross-section of 300 SRs from the National Library of Medicine’s Core Clinical Journals subset of journals published from 2009 to 2011 [11], but almost twice as quick as a cross-section of 129 SRs published in nursing journals in 2014, where the median time from search to publication online was around 15 months [18].

Interestingly, the small number of living or rapid SRs in our study were not likely to be published any quicker (median 91 vs 92 days); only preprints offered a considerably shorter time to publication (median 37 days). Thus an optimal solution for authors wanting to rapidly disseminate their reviews, while avoiding editorial delays, is to publish as a preprint when preparing their manuscript for submission to a journal (or to submit to journals that automatically make submissions available as preprints). As an added incentive to researchers, the visibility of preprints is increasing following Elsevier’s announcement in November 2021 of the inclusion of both MedRxiv and BioRxiv records in Embase. PubMed currently only includes preprints developed with U.S. National Institutes of Health support, which likely excludes the vast majority of systematic reviews.

Despite the relatively quick turnaround in conduct and subsequent publication of COVID-19 SRs, the question of the utility of these reviews remains, especially for reviews assessing the effects of potential treatments for COVID-19 [20]. The popularity of preprints [21, 22] and the proliferation of living reviews [23] have been notable features of the pandemic, and are an attempt to speed up the transfer of information. But even these approaches (in the case of living and rapid reviews) can still seem sluggish. Online platforms on the other hand, such as COVID-NMA.com, that are updated in near real-time offer an alternative way of providing up-to-date evidence synthesis [24].

Our finding that the number of included studies in the COVID-19 SRs was almost twice the number included in the sample of non-COVID-19 SRs (median 23 vs 12) should be interpreted cautiously. Many COVID-19 SRs, particularly those conducted early in the pandemic, tended to include a high proportion of case reports and case series rather than larger, more robust comparative studies. This likely explains why the median number of studies included in the COVID-19 SRs decreased from July 2020 to January 2021, despite the marked increase in COVID-related publications. The COVID-19 sample also included reviews of all types rather than solely effectiveness studies that comprised the non-COVID-19 sample.

### Strengths and limitations

While several research studies have investigated other aspects of reporting of COVID-related systematic reviews, including submission times and methodological quality, this is the first study, to the best of our knowledge, to look specifically at the reporting of search date information. Our sample was reasonably large and included a broad cross-section of COVID-19 systematic reviews addressing different types of questions, as well as including preprints, rapid and living reviews. The focus on the reporting of the search date means we are unable to comment on other aspects of the search, such as the range of sources consulted or the reporting of the search methods. A further limitation is that since the 2 months covered by our sample both fall within the first 12 months of the pandemic, it may not be reasonable to extrapolate these findings to later periods in the pandemic, particularly for the time from search to publication which had already increased from July 2020 to January 2021. Finally, the search used to derive the COVID-19 and non-COVID-19 samples differed. The search for the non-COVID-19 sample explicitly included terms for meta-analysis (in addition to the PubMed systematic review filter), while the COVID-19 sample relied solely on the PubMed SR filter. This is unlikely to affect our findings in relation to the search date reporting. The impact on the time to publication is less certain and context-specific—reviews with meta-analyses may or may not be quicker to complete than other forms of synthesis.

## Conclusions

Our study found that over four in 10 systematic reviews related to COVID-19 failed to provide any information in the abstract about when the search was conducted, and one in 17 failed to report this information anywhere in the review. In pandemics when evidence is accruing rapidly and users are seeking the most up-to-date syntheses of studies, it is incumbent on authors to report this information in the abstract. Journals also have a role in ensuring this information is available, in accordance with long-established reporting guidelines. Our finding of a median difference of 91 days (3 months) between the search date and publication of the review online (or 37 days for reviews first published as preprints) indicates that authors and journals greatly improved the timeliness of reviews early in the pandemic. However, even with these shortened timelines, the usefulness of traditionally published reviews to guide decision-making may still be limited given the rapid acceleration in published studies.

## Supplementary Information

**Additional file 1.**

## Data Availability

Data included in this study were extracted from published systematic reviews and can be made available on reasonable request.

## Abbreviations

IQR: Inter-quartile range
PRISMA: Preferred Reporting Items for Systematic review and Meta-Analyses
SR: Systematic review
