# Supplementary material for: Are COVID-19 systematic reviews up to date and can we tell? A cross-sectional study
Source: Syst Rev. 2023 May 18;12:85. doi: 10.1186/s13643-023-02253-x (PMC10193307; doi:10.1186/s13643-023-02253-x)
Supplement: Supplementary file 1 — Additional file 1. [file 13643_2023_2253_MOESM1_ESM.docx]

**Additional File 1: Are COVID-19 systematic reviews up to date and can we tell? A cross-sectional study**

**Appendix** – **Search strategies**

**For SRs added to PubMed in July 2020**:

(COVID-19[NM] OR "severe acute respiratory syndrome coronavirus 2" OR covid* OR sars* OR 2019nCoV OR coronavirus OR coronavirus infections OR "corona virus") AND systematic[SB] AND 2020/07:2020/07[EDAT] (Search run on 16 August 2000)

**For SRs added to PubMed in January 2021**:

((COVID-19[MeSH Terms] OR coronavirus* OR coronovirus* OR "corona virus" OR coronavirinae* OR COVID OR COVID-19 OR COVID19 OR sars* OR "severe acute respiratory syndrome*") NOT protocol[Title]) AND systematic[SB] AND 2021/01:2021/01[EDAT] (Search run on 10 February 2021)

**For the random sample of non-COVID-19 SRs added to PubMed in November 2020**:

(meta-analysis[PT] OR meta-analysis[TI] OR systematic[SB]) AND 2020/11/02:2020/12/02[EDAT] (Search run on 3 December 2020)
